# Lithium Chloride Shows Effectiveness against the Poultry Red Mite (*Dermanyssus gallinae*)

**DOI:** 10.3390/insects13111005

**Published:** 2022-11-01

**Authors:** Balázs Kolics, Éva Kolics, Izabella Solti, Zsuzsanna Bacsi, János Taller, András Specziár, Kinga Mátyás

**Affiliations:** 1 Festetics Bioinnovation Group, Institute of Genetics and Biotechnology, Georgikon Campus, Hungarian University of Agriculture and Life Sciences, H-8360 Keszthely, Hungary; 2 Institute of Agricultural and Food Economics, Georgikon Campus, Hungarian University of Agriculture and Life Sciences, H-8360 Keszthely, Hungary; 3 Balaton Limnological Research Institute, H-8237 Tihany, Hungary

**Keywords:** lithium chloride, contact effect, poultry red mite

## Abstract

**Simple Summary:**

The poultry red mite (*Dermanyssus gallinae*) is the major pest of poultry and a vector for many animal and human pathogens. Control is limited, and alternative substance efficacy may imply resistance and inconsistency problems. As a consequence of this, there is an increasing demand for alternative substances. In this pilot study, we report for the first time that lithium chloride, a currently promising potential miticide for *Varroa* mite (*Parasitiformes*) control in bees, showed effectiveness against poultry red mite in vitro. However, further comprehensive studies are needed to reveal whether lithium compounds can be considered an alternative miticide to control *Dermanyssus gallinae*.

**Abstract:**

The poultry red mite (*Dermanyssus gallinae*) is the main pest of poultry, causing severe problems by being a vector of several animal and human pathogens. The number of miticides is few, and their efficacy in practice implies problems of residues and resistance; therefore, the demand for a new and safe agent is constant. The present publication investigated the effectiveness of lithium chloride under in vitro conditions on poultry red mites. This chemical currently appears to be one of the most promising alternatives to study amongst potential applicants to treat varroosis, a fatal disease of honey bees. In Experiment I, the previously used experimental doses (5.52 M, 2.76 M, 1.38 M) on *Varroa* mites confirmed their in vitro activity on the poultry red mite. Three event times (uncontrolled movement, immobilisation and death) were recorded to base the response to treatment for each concentration. In Experiment II, the LD 50 value was calculated, i.e., the value at which 50% of the mites were killed by the treatment. This Experiment showed that the LD50 of lithium chloride = 0.265 M in the poultry red mite. It is to note that the study remained restricted to in vitro confirmation of lithium chloride’s effectiveness on the parasite. Thus, further extensive studies are needed to decide whether it has any relevance in practice against *D. gallinae*, and also to assess potential residue problems that could affect poultry products.

## 1. Introduction

Poultry red mite (*Dermanyssus gallinae*) has been a severe threat to animal health and welfare for decades, adversely affecting egg production and having significant public health implications. In infected animals, the first clinical symptom is anaemia due to subacute, recurrent mite bites. In extreme cases, *D. gallinae* infestation may be so intense that chicken may die of severe anaemia [1,2,3]. Mite infestation results in aggressive feather pecking and cannibalistic behaviour increased feed and water consumption, and thus deterioration of the general health status of the animals [4,5,6,7].

In addition to its direct impact on blood-sucking parasitism, *D. gallinae* is considered a vector for several avian viral and bacterial pathogens and can be a reservoir for numerous pathogens [5,8,9,10,11,12,13,14,15]. These have recently included Lyme borreliosis and avian influenza A virus [8,15]. Of the human pathogens, *Salmonella enteritidis* should be highlighted as having the highest global mortality rate among human zoonotic diseases [10,16]. The majority of cases are foodborne, and poultry products are the most common source of the disease [11,13]. In addition, *D. gallinae* infection is increasingly responsible for human dermatological lesions, notably gamasoidosis, especially in people living or working near poultry farms [8]. The disease is underdiagnosed [9], with an actual prevalence higher than is generally assumed [17]. Persistence in a fasting state for prolonged periods increases the vector’s role in pathogen maintenance [18].

Poultry infestation affects all types of production, from the backyard or organic farms to more intensive, improved cage or shed farming [19]. A recent epidemiological review found that 83% of European farms are infected, and this prevalence reaches 94% in the Netherlands, Germany and Belgium [20]. Mite damage is expected to increase due to recent changes in legislation on poultry production [1,16,17,21,22,23], climate change [24] and increased miticide resistance. The withdrawal of many miticides from international markets due to safety concerns and the persistent lack of new effective control methods has exacerbated the occurrence of *D. gallinae* in Europe. The need for effective and safe control of mites is therefore ongoing. Currently, there are a very limited number of active substances available for the treatment of mite infestations [19,25], and in addition, they show little or no efficacy against mite eggs, i.e., they do not prevent the recurrence of the pest population. The main active substances belong to the group of carbamates (carbaril, methomyl, propoxur), organophosphates (dichlorphos, phenitrothion, chlorpyrifos, diazinon) and pyrethroids (cihalothrin). The use of some of these (e.g., organophosphates) is already limited due to resistance issues [25,26].

Furthermore, the treatment of *D. gallinae* can pose critical risks to food safety and cause resistance development as a consequence of subtherapeutic doses [27,28,29,30]. Alternatively, used silica-based compounds and essential oils carry inconsistent efficacy and pose serious safety risks to the user and animals [5]. Developing new vaccine-based control strategies is promising; however, their efficacy is currently unsatisfactory [31,32]. For the above reasons, there is a continuous demand for new, lower residue risk and nature-identical insecticides.

Also belonging to *Parasitiformes and* order *Mesostigmata*, mite *Varroa destructor* is another important pest in agriculture. Although it has no human implications, it is also considered a viral vector. The resistance to different agents and the hidden lifestyle of reproductive individuals makes it the greatest challenge to apiculture and threatens the stability of pollination. Controlling the pest is challenging, and the need for new active substances is constant. Alongside novel approaches for the treatment of varroosis, lithium salts have been shown to be effective in vitro for eradicating *V. destructor* [33]. In addition to its systemic mechanism of action, a strong contact effect was confirmed to contribute to its high varroacidal efficacy [34]. In situ, it has outperformed oxalic acid, a widely used alternative to synthetic miticides to control varroosis of the honey bee. The *Varroa* mite and the poultry red mite share numerous common features of parasitism and result in similar impacts on resistance and residual-related issues. Lithium chloride might represent a practical, relatively inexpensive and potential alternative in apiculture worth further investigation [35]. In addition, preliminary results on chemical residues suggest that the risk of residues is expected to be low in beekeeping use [35,36]. However, for the moment, lithium is registered as a veterinary product only in Serbia [37].

Although it may be a promising miticidal agent in apiculture, the miticidal effectivity of lithium has not yet been reported on other taxa in the literature. Since problems similar to that of *V. destructor* arise concerning poultry red mite, we aimed to test whether the miticidal effectiveness of lithium may be extended to *D. gallinae*.

## 2. Materials and Methods

### 2.1. Mites

The mites were of mixed age, sex, and origin collected in July 2021, from poultry farms (Dabronc N: 47.0333, E: 17.1667, Cserszegtomaj N: 46.80613300, E: 17.23126210 and Miháld N: 46.265999, E: 17.076000 Hungary). Individuals were held at 24 ± 1 °C and 55 ± 5% relative humidity under a photoperiod of 16 h:8 h (light/dark) in a closed container with mesh prior to bioassays. Mites were tested within two days after collection. A total of 732 individuals were tested (Table 1).

### 2.2. Experimental Setup

Two experiments were conducted. Experiment I. tested the dynamics of the response of mites immersed in an aqueous lithium chloride solution of the following concentrations: 5.52 M, 2.76 M, and 1.38 M, similar to those applied in an earlier experiment with lithium chloride against the mite parasite of the honey bee [34]. The first recorded event was the onset of tremorous, uncontrolled movements. The second event was recorded when the mite could no longer change position but was still moving in one place, called the immobility state. Subsequently, the time of death was recorded as the third event.

Experiment II. was conducted to determine the concentration (LD50) at which lithium chloride kills half of the tested mites. To establish a concentration-response relationship, the mortality of mites was tested at ten concentrations: 5.520 M, 2.760 M, 1.380 M, 0.690 M, 0.345 M, 0.173 M, 0.086 M, 0.043 M, 0.014 M and 0.000 M (control).

Sample sizes are specified in Table 1.

### 2.3. Immersion Test

The response of mites to lithium chloride was investigated using the immersion method following Thomas et al. [38] with slight modifications. Each mite was immersed in a 1.5 mL Eppendorf tube containing 1 mL lithium chloride solution. The tubes were flipped up and down ten times for 10 s (one turn per second) in the bioassay. Subsequently, they were then placed on a filter disc (Sartorius, d = 150 mm, Grade: 1292). Dead specimens were discarded, leaving only the viable, undamaged specimens to examine, then placed onto Petri dishes, sealed, and held under laboratory conditions.

The control treatment was also carried out with an aqueous solution, and all treatments were replicated three times.

### 2.4. Statistical Analysis

In Experiment I., the elapsed time from the immersion was analysed separately for each event. Abbott’s formula [39] was applied to compute mortality rates. For the statistical analysis, data were LOG_10_(x + 1) transformed; then normality testing was carried out by the Jarque-Bera method; all group’s data were normally distributed (*p* > 0.05). Statistical differences were analysed with one-way ANOVA for each event separately. The homogeneity of variances was justified by Levene’s test (*p* > 0.05) for the log-transformed values of time to immobility and death but not for time to uncontrolled movement. Therefore, pairwise differences were analysed with Tukey’s post hoc test for time to immobility and time to death, with homogeneous variances, and Tamhane’s post hoc test for the time to uncontrolled movements, with inhomogeneous variances [40].

A classic logistic growth curve (also known as the Pearl-Reed logistic curve) was fitted to the Abbott corrected mortality rate data for each treatment, of the form y = K/(1 + b × e^−cx^) with K = 100, and y representing the mortality rate and x the exposure time. Parameters c and b define the position and slope of the fast-growth section of the curve, with x = lnb/c leading to y = K/2. This means that higher c values make the curve steeper, while with higher b values, the 50% value (LD50 stage) is reached later at higher × values.

The statistical tests were computed by SPSS 22.0 software [41].

In experiment II., Hill model (i.e., 4 parameters logistical) [42] was fitted to mortality data of mites by lithium chloride concentrations, and the LD50 value was calculated based on the nett effect of the tested substance (i.e., mortality above the control level) by using the calculator of AAT Bioquest Inc. (Pleasanton, CA, USA) [43].

## 3. Results

**Figure 1:**
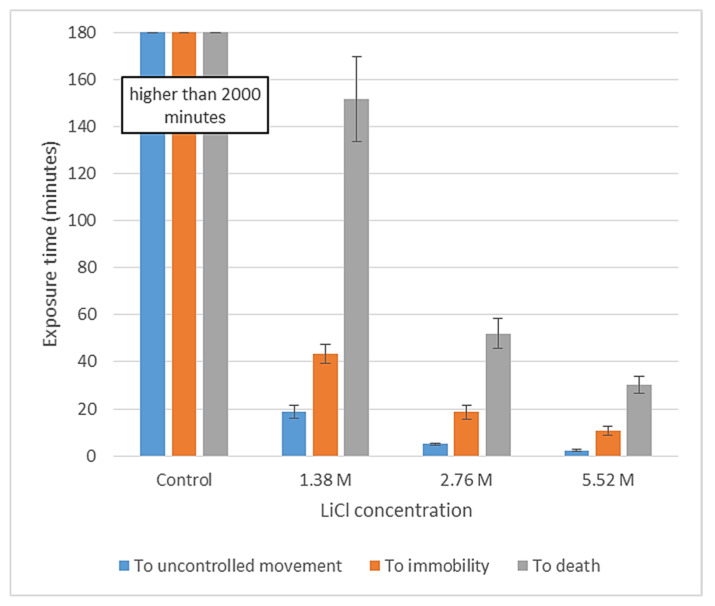
Mean exposure time from immersion to occurrence of an event, with error bars denoting ± SE (standard error) of the mean, by LiCl concentration. For statistics based on LOG_10_(x + 1) transformed data, see Table 2.

In Experiment I., differences were detected between all concentrations for the mean exposure times to each recorded event (i.e., uncontrolled movement, immobility, death), with higher concentrations leading to shorter mean times (Figure 1 and Table 2).

**Figure 2:**
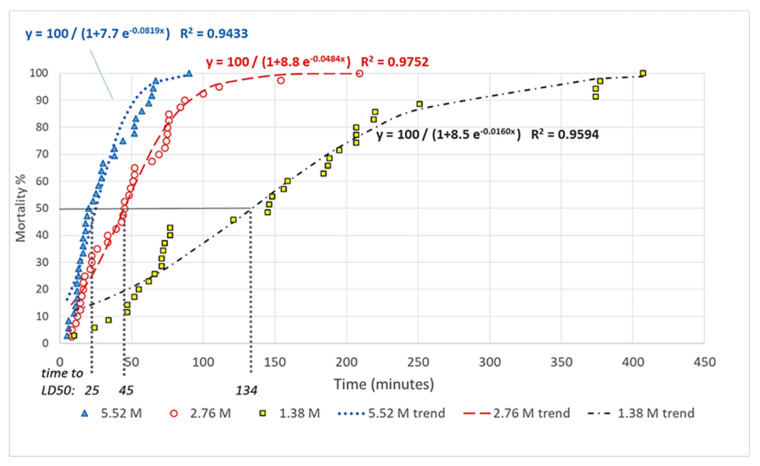
Abbott corrected mortality rates and fitted trends for the three LiCl-concentrations.

Classic logistic growth curves fitted to the Abbott corrected mortality rate data by treatment concentrations are shown in Figure 2. All R2 values are above 0.94, indicating good fits of the estimated trend lines. With higher doses of treatment, the speed of mortality increases. The exposure times needed to LD50 decrease with higher concentrations relatively fast; when doubling the concentration from 1.38 M to 2.76 M, the exposure time decreases to one-third (from 134 to 45) and then doubling the concentration from 2.76 M to 5.52 M results in nearly halving the exposure time again (from 45 to 25). Table 3 gives the elapsed time elapsed for the three concentrations till the 50% (LT50), and 90% (LT90) death rates, respectively, clearly showing the effectiveness of the higher concentrations.

**Figure 3:**
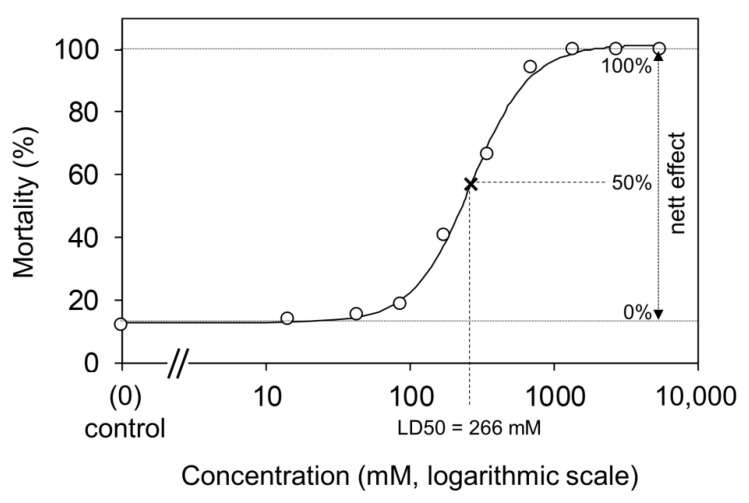
Concentration-response curve and LD50 concentration value for lithium chloride in poultry red mite. Note that LD50 value was calculated based on the net effect of the tested substance (i.e., mortality above the control level).

Experiment II. revealed that LD50 = 0.265 M for lithium chloride in poultry red mite (Figure 3).

## 4. Discussion

In the present study, we have demonstrated that lithium chloride shows effectiveness against poultry red mite in vitro. All the treated groups developed detectable signs of lithium poisoning, and the time courses of intoxication were inversely proportional to concentration for all treatments. These results support recent findings with lithium chloride as an effective potential acaricide in beekeeping [33,34,44,45], indicating that the potential applicability of lithium salts for mite infections in cultured animals might deserve further testing.

The risk of residues, however, is critical for using the microelement as an acaricide. Results so far indicate that the residues of lithium in honey as a consequence of anti-*Varroa* treatment are not alarming [35,36], supported by the fact that many foods may contain lithium as a trace element [46,47]. Foods of animal origin are generally rich in naturally occurring micro elemental lithium, also involving milk, poultry meat and eggs (>7000 μg dry matter) [46,47,48,49]. Furthermore, chemical residues can be judged as residues of a trace element, the daily intake of which in the milligram range can prevent progression or delay the onset of chronic diseases and may elicit a positive effect on life expectancy in general, mainly by suppressing the function of the glycogen synthase kinase-3 (GSK3) enzyme [50,51]. Moreover, 1 mg of lithium per day has been proposed as a micronutrient supplement [48], with a recommended daily intake of 1.0 mg lithium/day for 70 kg adult [52]. However, in higher amounts than trace element levels, lithium is a common alternative to treating bipolar disorder in human medicine with side effects on the kidney. It should be noted that the lithium intake, in this case, is several times the range of the microelement (~170 mg Li^+^/day).

Present results for the first time indicate an interesting aspect of lithium as having acaricidal effectiveness on the most important mite parasite of poultry, with LD50 effect at 0.265 M concentration. Still, comprehensive research is required to uncover if there would be any effective method of administering lithium in situ, to know whether lithium chloride may have practical relevance. Furthermore, taking that lithium overdose may pose the risk of building chemical residues, and extensive studies on the kinetics of lithium are needed in poultry products in situ. 

## Figures and Tables

**Table 1:** The number of mites tested (N) in Experiments I. and II.

Licl Concentration	Experiment I. N	Experiment II. N
5.520 M	50	32
2.760 M	50	33
1.380 M	50	29
0.690 M		66
0.345 M		56
0.173 M		111
0.086 M		82
0.043 M		33
0.014 M		22
0.000 (CONTROL)	50	68
TOTAL	200	532

**Table 2:** Mean ± SE (standard error) for the log-transformed exposure times by concentration and results of the ANOVA statistics.

Licl Concentration	Uncontrolled Movement	Immobility	Death
1.38 M	1.183 ± 0.051 a	1.5775 ± 0.045 a	2.062± 0.061 a
2.76 M	0.750 ± 0.027 b	1.1851 ± 0.043 b	1.602 ± 0.054 b
5.52 M	0.459 ± 0.036 c	0.9275 ± 0.055 c	1.388 ± 0.052 c
ANOVA	F(2,108) = 87.549 *p* < 0.05	F(2,108) = 45.446 *p* < 0.05	F(2,108) = 36.626 *p* < 0.05

Note: (a, b, c) indicate concentrations significantly different from others, according to the Tukey and the Tamhane post-hoc tests. F shows the F-test statistic value to compare with the theoretical F-distribution, high F-values indicating significant differences between the concentrations. *p* values are the probability of erroneously concluding significant differences between the concentrations.

**Table 3:** Time to the death of 50% (LT50) and 90% (LT90) of animals (in minutes).

Licl Concentration	LT50 (Minutes)	LT90 (Minutes)
5.52 M	25	64
2.76 M	45	87
1.38 M	134	312.5
